# Evaluation of the Toxicity of *Pradosia huberi* Extract during the Preimplantation in Wistar Rats

**DOI:** 10.1155/2013/294172

**Published:** 2012-12-20

**Authors:** Aldeíde de Oliveira Batista Rocha, Liliane de Queirós Sousa, Clélia de Alencar Xavier Mota, Elane Cristina S. Santos, Margareth de Fátima Formiga Melo Diniz, Marcelo Sobral da Silva, Martina Bragante F. Pimenta, Rita de Cássia da Silveira e Sá

**Affiliations:** ^1^Biotechnology Center, Federal University of Paraíba, P.O. Box 5009, 58051-970 João Pessoa, PB, Brazil; ^2^Department of Pharmaceutical Sciences, Federal University of Paraíba, P.O. Box 5009, 58051-970 João Pessoa, PB, Brazil; ^3^Department of Pathology and Physiology, Federal University of Paraíba, P.O. Box 5009, 58051-970 João Pessoa, PB, Brazil

## Abstract

The treatment during the embryonic preimplantation phase of Wistar rats with the *Pradosia huberi* extract did not interfere with the water and feed consumption, as well as upon the body-weight gain. However, it has expressed a decrease of the uterine implant number, followed by the preimplantation losses at all applied doses (1.22, 6.1, and 30.5 mg/kg), and the number of embryonic resorptions in the two highest doses (6.1 and 30.5 mg/kg). After the organ weighing (hypophysis, ovaries, and uterus), only the relative weight of the hypophysis was raised at the different doses (1.22, 6.1, and 30.5 mg/kg). It was concluded that the hydroalcoholic extract of *Pradosia huberi* compromises the reproductive ability during the embryonic preimplantation phase, suggesting a possible toxic effect upon the reproductive system of Wistar rats.

## 1. Introduction

The occurrence of biologically adverse effects upon the female reproductive system which affect the fertility and the reproductive ability can be expressed as changing the production and transport of gametes, in the reproductive cycle, endocrine system, sexual behavior, gestation, parturition and lactation, or alterations in other functions which depend of the integrity of the reproductive system [1, 2].

 Numerous plants are known by presenting potential toxic effects upon the mammals' reproduction such as previous placenta, postterm pregnancy, premature uterine contractions, and even abortion [3, 4]. *Pradosia huberi* Ducke (Sapotaceae) is popularly known as “casca doce” or “pau doce” in the folk medicine [5] and it is widely used in the treatment of ulcers and gastritis due its anti-inflammatory properties upon the gastrointestinal system [6]. Phytochemical studies of the *P. huberi* bark revealed the presence of the following flavonoids: 2.3-dihydromyricetin 3-*α*-L-rhamnoside, astilbin, engelitin and dihydromyricetin-2.3 [7], and 2.3-dihydromyricetin-3-O-rhamnoside acetate and 2.3-dihydromyricetin-7-O-rhaminoside acetate [8]. Despite flavonoids having a wide representation in the Plantae kingdom associated to their broad therapeutic potential [9, 10], numerous surveys have revealed and alerted the population about their possible systemic toxicological effects, including the reproduction [11]. The glabridin—isolated flavonoid of *G. glabra*—showed antiestrogen activity, compromising the reproductive hormone physiology [12]. Besides that, the *Ginkgo biloba *extract which also has flavonoids in its composition induced apoptosis in the embryonic stem cells of rats [13].

 Considering the vast use of the studied plant associated to the presence of substances with possible deleterious activity upon the reproductive process, this study aimed to evaluate the effects of hydroalcoholic extract of *P. huberi* during the uterine preimplantation time of Wistar rats.

## 2. Material and Methods

### 2.1. Animals and Experimental Groups

 Adult Wistar rats (*Rattus norvegicus *Berkenhout, 1769) were used, aged 90 days and weighing approximately 200–250 g, and were provided by the vivarium of the Biotechnology Center of UFPB. The rats were housed in polyethylene cages and kept under controlled temperature conditions (21 ± 2°C), obeying to a light-dark cycle of 12 hours each, without medication and with free access to pelleted feed of Purina and drinking water during the whole experimental time.

 This study was approved by the Ethics Committees on Animal Research of the CB/UFPB, number 0205/10.

 The animals were randomly distributed into four experimental groups, three being treated and one a control, containing 16 females each.

### 2.2. Botanical Material and Extract Preparation

The bark of *Pradosia huberi* was collected in the city of Porto Grande, AP, Braul, where a voucher specimen number 012519 was deposited in the Herbarium of Amapá (HAMAB) of the Instituto de Pesquisas Científicas e Tecnológicas do Estado do Amapá-IEPA (Institute of Scientific and Technological Research of Amapá). The plant material was prepared in the Chemistry Lab of IEPA. In order to obtain the hydroalcoholic extract (HAE) the plant material was held under controlled-temperature kiln drying (38°C), with EtOH mixture (70%): H_2_O at room temperature (25–30°C) for 72 hours. Later, the obtained mixture was filtered and concentrated under vacuum in a rotary evaporator in a temperature of 50°C until obtaining the hydroalcoholic extract [14]. Then the HAE was diluted in distilled water to obtain solutions in appropriate concentrations for correct administration of doses used in this study (1.22, 6.1, and 30.5 mg/kg) [15], which were based on prior studies that have evaluated the antiulcerogenic activity of *P. huberi* in rats and mice [6].

### 2.3. Evaluation of the Reproductive Toxicity

#### 2.3.1. Mating

 The mating system was polygamous, in which three maiden females were placed in the box of each male, where they were kept during the dark cycle. Next morning, the animals were separated and the verification of pregnancy was performed by the presence of sperm in a vaginal smear, carried out daily between 7 and 8:0 AM and analyzed in a 10x optical microscope. Twenty-four hours after the observation of the presence of sperm in the vaginal smear was set as the first day of pregnancy. The mating was repeated until a sufficient number of progenitor cells for performing the experiments was obtained [16, 17].

#### 2.3.2. Exposure during the Preimplantation and Tests of Reproductive Toxicity

 The pregnant females (*n* = 64) were treated with HAE of *P. huberi* from the first to the seventh day of pregnancy, comprising the phase of embryonic preimplantation in rats which occurs from five to six days after fertilization [18]. During this period, the animals were monitored for the analysis of the maternal systemic toxicity, such as irritability, seizures, ataxia, sedation, diarrhea, cyanosis, hair loss, and deaths, besides the water and ration consumption and weight gain [19]. 

 On the eighth day of pregnancy, females were sacrificed by an excessive dose of ketamine (König) and the reproductive variables were investigated through the analysis of the following parameters: number of corpora lutea on ovaries; number of uterine implants; number of resorptions; gestation index ((number of pregnant females/number of inseminated females) × 100) and the index of preimplantation losses ((number of corpora lutea − number of implants/number of corpora lutea) × 100) [1, 20–22].

In addition, the absolute and relative weight ((organ weight/body weight) × 100) of the hypophysis, left and right ovaries, and the pregnant uterus were recorded [14, 23], and the dosage of serum levels of progesterone was performed by the enzyme reaction method by microparticles (automatic biochemical analyzer-Axsym).

#### 2.3.3. Statistical Analysis

 The data were analyzed by the variance analysis (ANOVA) and the differences among the groups were determined by Tukey test. The variables listed as index were analyzed by chi-square test, excepting the relative weight of the organs which was also analyzed using ANOVA followed by Tukey test.

 All data obtained were statistically analyzed by the program GraphPad Prism, version 4.0 (GraphPad Software Inc., San Diego, CA, USA). The difference among the groups was considered as significant for *P* < 0.05.

## 3. Results

### 3.1. Exposure during the Preimplantation and Tests of Reproductive Toxicity

#### 3.1.1. Systemic Signs of Maternal Toxicity

**Figure 1:**
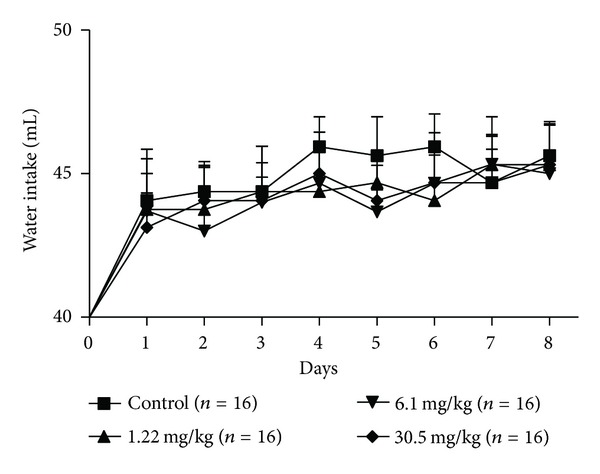
Water intake of female rats treated with the hydroalcoholic extract of *P. huberi*, during the preimplantation. The values are expressed as mean ± S.E.M. *P < 0.05 versus control group. ANOVA followed by Tukey test. The *n* represents the number of progenitors.

**Figure 2:**
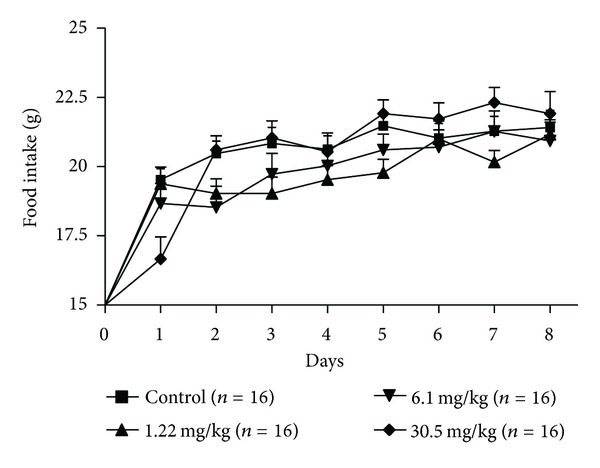
Food intake of female rats treated with the hydroalcoholic extract of *P. huberi*, during the preimplantation. The values are expressed as mean ± S.E.M. *P < 0.05 versus control group. ANOVA followed by Tukey test. The *n* represents the number of progenitors.

**Figure 3:**
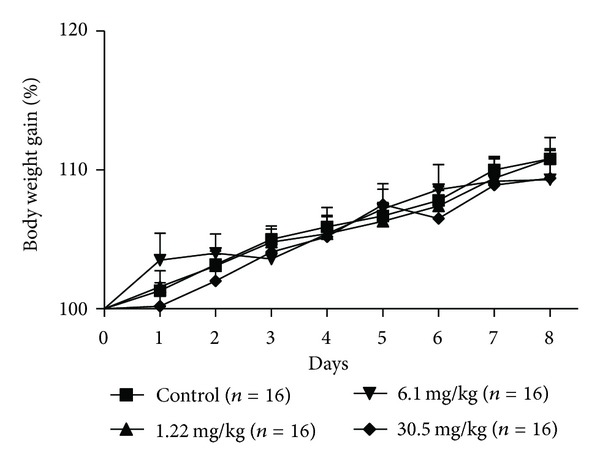
Body weight gain of female rats treated with the hydroalcoholic extract of *P. huberi* during the preimplantation. The values are expressed as mean ± S.E.M. *P < 0.05 versus control group. ANOVA followed by Tukey test. The *n* represents the number of progenitors.

 During the seven days of pregnancy, a period which corresponds to the preimplantation, the rats daily treated with the hydroalcoholic extract of *P. huberi* showed no symptomatic signs of systemic toxicity, such as irritability, seizures, ataxia, sedation, diarrhea, cyanosis, and hair loss; also no deaths were reported, as well as no change of water consumption (Figure 1) and food (Figure 2) swallowed by females treated when compared to the control groups. Similarly, the weight development of animals exposed to the extract from the first to the seventh day of pregnancy showed a normal growth pattern among the groups treated with doses of 1.22, 6.1, and 30.5 mg/kg when compared to the control group (Figure 3).

#### 3.1.2. Reproductive Variables

 As shown in Table 1, the daily exposure to the extract significantly decreased the number of uterine implantations, being followed by the increase of the preimplantation rates in the three applied doses (1.22, 6.1, and 30.5 mg/kg) and the index number of resorptions for the animals which received the two highest doses (6.1 and 30.5 mg/kg). The other variables were not changed.

#### 3.1.3. Body Weight and Relative and Absolute Weights of Organs

According to the data presented in Table 2, the rats treated during preimplantation showed no significant difference in their body weight during sacrifice time, nor in the absolute weight of organs (hypophysis, ovaries, and uterus); however, the relative weight of the hypophysis gland expressed statistically significant increase in the three applied doses (1.22, 6.1, and 30.5 mg/kg) when compared to the control group. The other organs (ovaries and uterus) did not express changes in their relative weights.

#### 3.1.4. Hormone Dosage

**Figure 4:**
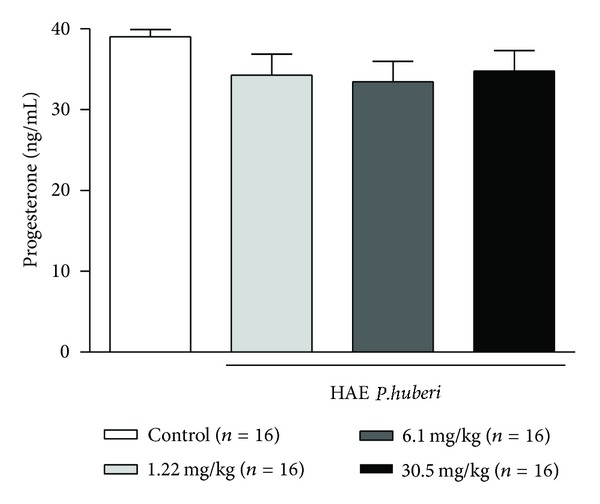
Progesterone dosage of female rats treated with the hydroalcoholic extract of *P. huberi*, during the preimplantation. The values are expressed as mean ± S.E.M. *P < 0.05 versus control group. ANOVA followed by Tukey test. The *n* represents the number of progenitors.

 The concentration of serum progesterone did not suffer significant change in female rats that received the extract in different doses: 1.22 mg/kg (34.4 ± 2.5), 6.1 mg/kg (33.4 ± 2.5), and 30.5 mg/kg (34.7 ± 2.5) when compared to the control group (39.0 ± 0.9) during the preimplantation (Figure 4).

## 4. Discussion

This study investigated the possible toxicological effects of hydroalcoholic extract of *P. huberi* upon the reproductive system of Wistar rats during the embryonic preimplantation time, since this is a very delicate stage which requires perfect coordination of physiological events for the maintenance and the success of pregnancy [24, 25]. In addition, the medicinal plants are a source of active compounds able to exert therapeutic and toxic activities [26]. For example, *P. huberi* also has gastroprotective activity [6] but revealed high toxicity, causing behavioral changes, decreased weight of organs, and death in rodents [27].

 Signs of systemic toxicity are defined from the reduction of body weight of animals, food and water consumption, and the outbreak of physical and behavioral changes, since the modification of such parameters reflects the toxic potential of a substance upon the organ systems, including the reproductive system [28]. Because this under hormonal influence of estrogen and progesterone interferes with the water and food ingestion, energy balance, fluid retention, and fat deposition by the female organism [29].

 In this study the animals treated with the hydroalcoholic extract of *P. huberi* in different doses (1.22, 6.1, and 30.5 mg/kg) during the preimplantation did not show symptomatic systemic signs of maternal toxicity, suggesting the nonphysiological commitment of the central and autonomous nervous system [30].

 The analysis of the reproductive variables goes through the ovaries' evaluation, which allows the investigation, besides its hormonal function, important reproductive indices by counting the number of corpora lutea which has direct relation to the amount of oocytes released during ovulation, allowing this way an analysis of the actual number of fertilized oocytes, besides thoughtful observation of the uterus in order to count the implantation sites and resorptions [1, 31].

 The daily treatment with *P. huberi* extract during the preimplantation presented evidence of reproductive toxicity, since the preimplantation losses were significantly increased, as indicated by data which were monitored by the decreasing of uterine implants in rats treated with different doses of extract (1.22, 6.1, and 30.5 mg/kg) and an increase in the number of resorptions for those animals which received the two highest doses (6.1 and 30.5 mg/kg). Such results suggest embryotoxicity, thus jeopardizing the reproductive capacity of females treated with the extract, to an order that caused loss of embryos at the development stage in the fallopian tubes [32].

 In a similar study, rats treated with the hydroalcoholic extract of *Baccharis trimera* and flavonoids isolated from this plant have expressed significant reduction in the implantation and an increase in the preimplantation loss rate, suggesting a relaxing effect on the smooth muscles of the fallopian tube, with interference in the transport of the blastocyst up to the uterus [33].

 In addition, the investigation of the toxic potential of a substance upon the reproductive system must include the dosage of serum levels of hormones involved in the homeostasis of the hypothalamic axis, hypophysis, and gonads [1]. First, the hypothalamus produces and secretes the GnRH which stimulates the hypophysis to release the FSH and LH; these, in turn, have a direct action upon the ovaries promoting follicular development and release of the oocyte [34].

 The animals treated with the hydroalcoholic extract of *P. huberi* had the relative weight of their hypophysis increased, when compared to the control group, suggesting a possible effect of the extract on the hypophysis function. Several studies have demonstrated the adverse effect of substances in the hypothalamic, pituitary, and gonadal axis. For example, the treatment of Wistar rats with bisphenol A (insecticide) in high doses increased the weight of the hypophysis and elevated the prolactin levels compared to castrated rats [35–37]. However, the increased weight of the hypophysis caused by using the extract of *P. huberi* did not present direct correlation with hormone production, because the serum levels of progesterone and the number of corpora lutea were not changed, demonstrating a non-antiestrogenic effect, at least regarding the ovarian production of progesterone. Through ovarian weight and number of corpora lutea it is possible to deduct indirectly the hormonal conditions related to maternal progesterone, because the ovary weight is directly proportional to the number of corpora lutea which is the largest structure found in this organ [38].

 Therefore, the analysis of the data obtained in this study indicates that the maternal hormone homeostasis, essential for embryonic development, of rats treated with the extract of *P. huberi* was not changed by inadequate levels of progesterone, whose reduction would undermine the viability of the embryo, by preventing the endometrium from being prepared to ensure the maintenance of pregnancy. However, the possibility of the extract of *P. huberi* being an endocrine disrupter cannot be ruled out, due to its interference of the embryonic preimplantation in Wistar rats, as previously evidenced by a reduction in the number of uterine implantations and increased resorptions. However, more studies should be carried out in order to monitor hormone production during the pregnancy period, because the success of a pregnancy requires a perfect physiological harmony among the hypothalamic, pituitary, and gonadal functions to ensure the transportation and integrity of the gamete and the zygote, and to enable the success of fertilization and embryo survival [39].

## 5. Conclusions

 Based upon the obtained results and under adopted experimental conditions, the treatment of Wistar rats with the hydroalcoholic extract of *P. huberi* during the preimplantation induced suggestive reproductive changes of toxicity on the reproductive system of Wistar rats. However, further studies are necessary to elucidate the mechanism of action behind the observed effects.

## Figures and Tables

**Table 1:** Reproductive variables of rats treated with the hydroalcoholic extract of *P. huberi *during the preimplantation.

Variables	Hydroalcoholic extract of *Pradosia huberi *			
	Control	1.22 mg/kg	6.1 mg/kg	30.5 mg/kg
Number of inseminated rats	16	16	15	16
Number of pregnant rats	15	11	12	13
Number of corpora lutea	6.4 ± 0.2	5.5 ± 0.4	5.8 ± 0.3	6.9 ± 0.4
Number of uterine implants	11.0 ± 0.1	6.6 ± 1.3*	8.2 ± 1.4*	8.7 ± 1.4*
Number of resorptions	0.1 ± 0.1	0.3 ± 0.2	1.0 ± 0.5*	1.5 ± 0.6*
Gestation index (%)	93.7	68.7	80.0	81.2
Preimplantation loss (%)	14.1	39.2*	28.7*	33.3*

Values are mean ± S.E.M. *P < 0.05 versus control.

Gestation index = (number of pregnant females/number of inseminated females) × 100.

Index of preimplantation loss = (number of corpora lutea – number of implants/number of corpora lutea) × 100.

**Table 2:** Body weight and absolute and relative weights of rats treated with the hydroalcoholic extract of *P. huberi *during the preimplantation.

Variables	Hydroalcoholic extract of *Pradosia huberi *			
	Control (*n* = 16)	1.22 mg/kg (*n* = 16)	6.1 mg/kg (*n* = 15)	30.5 mg/kg (*n* = 16)
Body weight (g)	252.3 ± 7.2	242.1 ± 9.7	236.8 ± 7.8	248.3 ± 5.5
Absolute weight (mg)				
Hypophysis	10.1 ± 0.1	11.3 ± 0.2	11.6 ± 0.3	11.9 ± 0.3
Right ovary	34.6 ± 0.6	31.7 ± 0.8	32.2 ± 1.1	31.1 ± 0.8
Left ovary	32.7 ± 0.8	31.4 ± 0.8	30.8 ± 0.9	32.1 ± 0.8
Uterus	783.6 ± 35.7	693.2 ± 39.0	690.0 ± 42.1	724.9 ± 58.9
Relative weight (%)				
Hypophysis	0.003 ± 0.0001	0.004 ± 0.0001*	0.005 ± 0.0002*	0.005 ± 0.0002*
Right ovary	0.01 ± 0.0004	0.01 ± 0.0004	0.01 ± 0.0007	0.01 ± 0.0004
Left ovary	0.01 ± 0.0006	0.01 ± 0.0006	0.01 ± 0.0006	0.01 ± 0.0004
Uterus	0.30 ± 0.002	0.24 ± 0.002	0.25 ± 0.003	0.26 ± 0.002

Values are mean ± S.E.M. *P < 0.05 versus control.
